# Regulation of pyruvate metabolism and human disease

**DOI:** 10.1007/s00018-013-1539-2

**Published:** 2013-12-21

**Authors:** Lawrence R. Gray, Sean C. Tompkins, Eric B. Taylor

**Affiliations:** Department of Biochemistry, Fraternal Order of the Eagles Diabetes Research Center, and François M. Abboud Cardiovascular Research Center, Roy J. and Lucille A. Carver College of Medicine, University of Iowa, 51 Newton Rd, 4-403 BSB, Iowa City, IA 52242 USA

**Keywords:** Pyruvate, Mitochondria, Mitochondrial pyruvate carrier (MPC), Metabolism, Human disease

## Abstract

Pyruvate is a keystone molecule critical for numerous aspects of eukaryotic and human metabolism. Pyruvate is the end-product of glycolysis, is derived from additional sources in the cellular cytoplasm, and is ultimately destined for transport into mitochondria as a master fuel input undergirding citric acid cycle carbon flux. In mitochondria, pyruvate drives ATP production by oxidative phosphorylation and multiple biosynthetic pathways intersecting the citric acid cycle. Mitochondrial pyruvate metabolism is regulated by many enzymes, including the recently discovered mitochondria pyruvate carrier, pyruvate dehydrogenase, and pyruvate carboxylase, to modulate overall pyruvate carbon flux. Mutations in any of the genes encoding for proteins regulating pyruvate metabolism may lead to disease. Numerous cases have been described. Aberrant pyruvate metabolism plays an especially prominent role in cancer, heart failure, and neurodegeneration. Because most major diseases involve aberrant metabolism, understanding and exploiting pyruvate carbon flux may yield novel treatments that enhance human health.

## Introduction

**Fig. 1:**
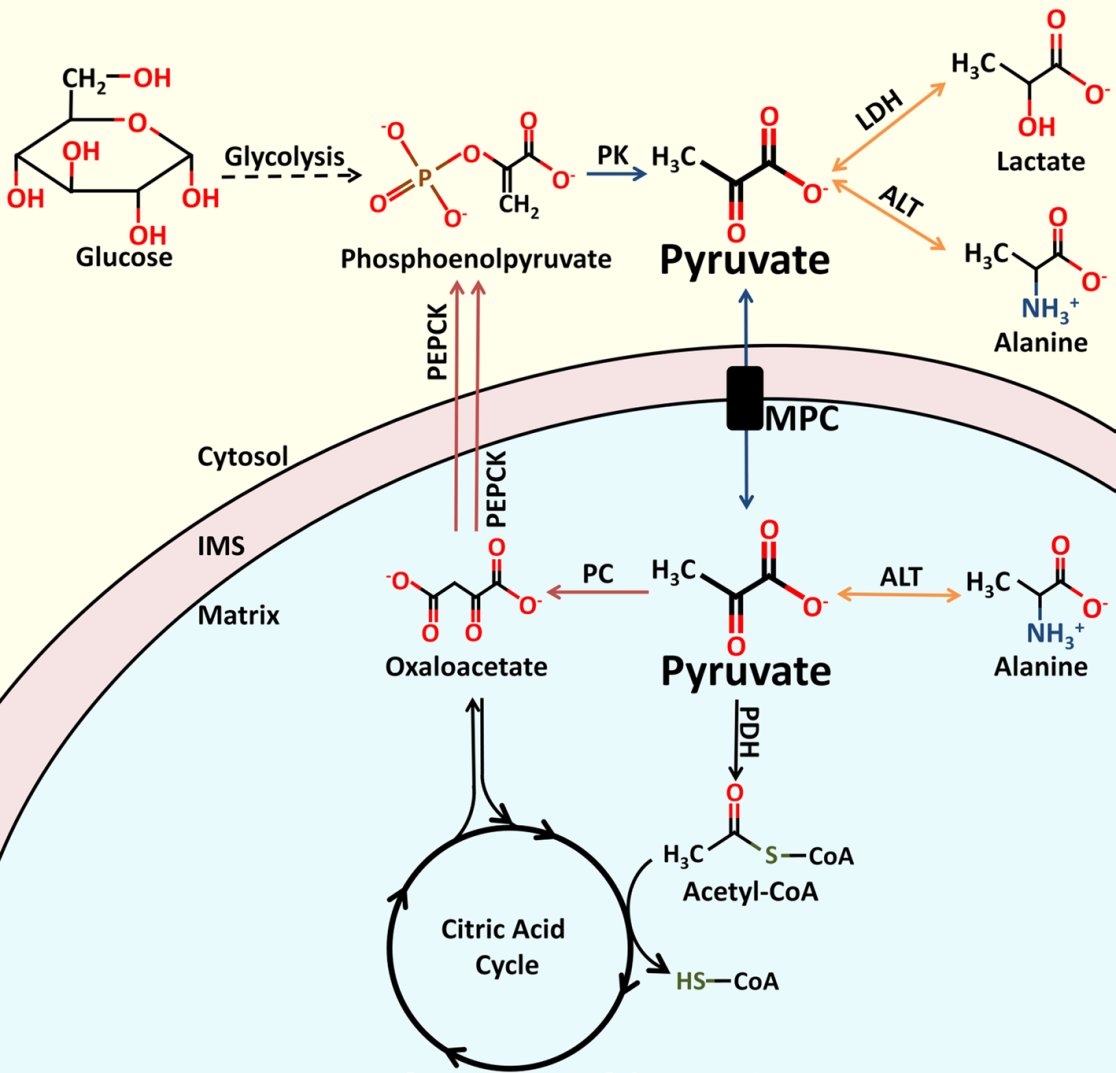
Enzymes involved in proximal pyruvate metabolism. Pyruvate plays an essential role in central carbon metabolism. Pyruvate is generated from several sources, including the oxidation of lactate, the transamination of alanine, or as the terminal product of glycolysis. Entry of pyruvate into the mitochondrial matrix is mediated by the MPC. Once in the matrix, pyruvate can be converted to acetyl-CoA or oxaloacetate. Oxaloacetate can enter the citric acid cycle to replenish intermediates, or be converted to phosphoenolpyruvate as part of the gluconeogenic pathway. Phosphoenolpyruvate can be formed from oxaloacetate by PEPCK within the mitochondria or within the cytoplasm. The molecular structures of pyruvate and related metabolites, as well the names of the enzymes involved in their catalysis, are shown. *PK* pyruvate kinase, *LDH* lactate dehydrogenase, *ALT* alanine aminotransferase, *MPC* mitochondrial pyruvate carrier, *PDH* pyruvate dehydrogenase, *CoA* Coenzyme A, *IMS* mitochondrial inner membrane space, *PEPCK* phosphoenolpyruvate carboxykinase

**Fig. 2:**
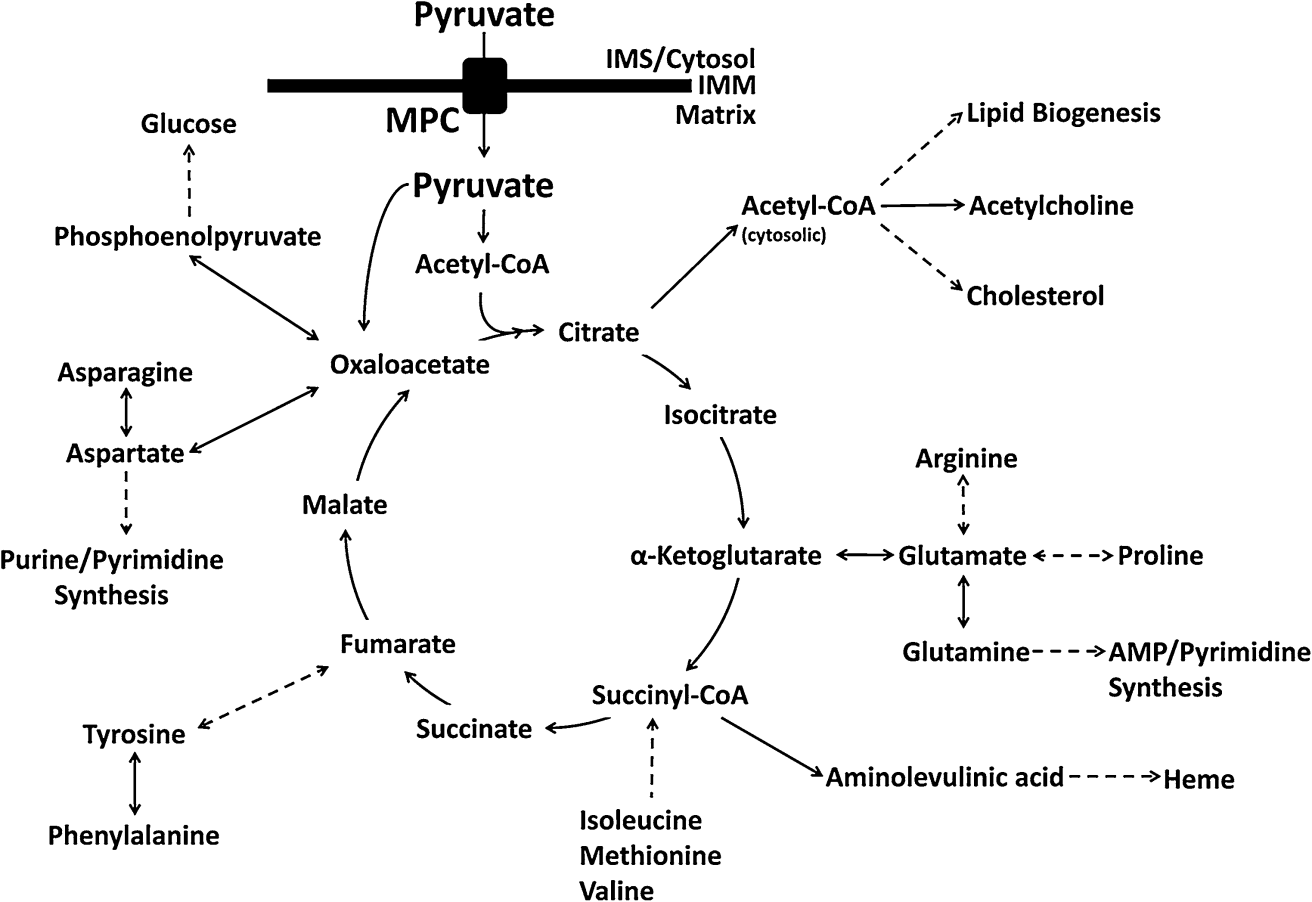
Pyruvate and citric acid cycle carbon flux. Pyruvate is the master carbon fuel input supporting overall citric acid cycle carbon flux. Pyruvate transits the inner mitochondrial membrane (IMM) through the mitochondrial pyruvate carrier (MPC) to reach the mitochondrial matrix. In the matrix, pyruvate carbon enters the citric acid cycle as citrate or oxaloacetate, depending on the need to replenish oxaloacetate. Numerous metabolic pathways intersect the citric acid cycle. The modulation of mitochondrial pyruvate flux balances for anaplerotic carbon entrance and cataplerotic carbon exit to ensure continued cycle flux. Disruption of mitochondrial pyruvate flux may subsequently disrupt carbon flux through any of the pathways intersecting the citric acid cycle

Pyruvate is a keystone molecule critical for numerous aspects of eukaryotic and human metabolism. Pyruvate is the end-product of glycolysis, is derived from additional sources in the cellular cytoplasm, and is ultimately destined for transport into mitochondria where it is the master fuel input undergirding citric acid cycle carbon flux (Fig. 1). Accordingly, pyruvate is critical for mitochondrial ATP generation and for driving several major biosynthetic pathways intersecting the citric acid cycle (Fig. 2).

Disruption in pyruvate metabolism, depending on the location or severity of the mutation, causes mild to severe disease (Table 1). Tissues with a high demand for ATP are most affected, with the nervous system being particularly vulnerable because of its predominate reliance on carbohydrate metabolism for ATP generation. Aberrant pyruvate metabolism may arise from mutations in any of the many genes coding for enzymes that regulate it. Most of these enzymes have been well studied for decades, yet additional critical aspects of pyruvate metabolism are just beginning to be understood. The mitochondrial pyruvate carrier (MPC), which serves as a highly critical link between cytosolic and mitochondrial pyruvate metabolism, was only recently identified [1, 2]. This review will discuss the enzymes regulating major aspects of pyruvate metabolism, their structures, and the biochemical bases for the reactions they catalyze, the roles dysfunctional forms play in causing human disease, and major diseases for which aberrant pyruvate metabolism is a prominent characteristic.

**Table 1:** Overview of enzymes involved in proximal pyruvate metabolism

Enzyme	Reaction	Metabolic deficiency symptoms	Incidence
Pyruvate dehydrogenase (PDH)	Pyruvate +NAD → CO_2_ + Acetyl-CoA + NADH	Neurodegeneration, lactic acidosis, hyperpyruvicemia, psychomotor retardation/developmental delay	Rare (350 + cases) [89, 90]
Lactate dehydrogenase (LDH)	Pyruvate + NADH ↔ lactate + NAD^+^	Myoglobinuria, elevate pyruvate levels, low endurance/exercise intolerance	1:1,000,000 [34, 294]
Pyruvate carboxylase (PC)	Pyruvate + ATP + CO_2_ → Oxaloacetate + ADP	Highly variable, depends upon classification (Types A, B, or C) May include lactic acidosis, developmental delay, and elevated proline and alanine levels	1:250,000 [127]
Pyruvate kinase (PK)	Phosphoenolpyruvate + ADP → Pyruvate + ATP	Hemolytic anemia, hyperbilirubinemia	1:20,000 [15]
Alanine aminotransferase (ALT)	Pyruvate + glutamate ↔ Alanine + α-ketoglutarate	Unknown (mild)	2.5:1,000 [48]
Mitochondrial pyruvate carrier (MPC)	Pyruvate_IMS_ ↔ pyruvate_Matrix_	Neurodegeneration, lactic acidosis, hyperpyruvicemia, psychomotor retardation	Very rare (2 cases) [1, 68]
Pyruvate dehydrogenase phosphatase (PDP)	P-PDH → PDH + Pi	Lactic acidosis, elevated pyruvate and alanine levels, exercise intolerance, hypotonia	Very rare (2 cases) [113, 114]
Pyruvate dehydrogenase kinase (PDK)	PDH + ATP → P-PDH + ADP	N/A	N/A

This table summarizes the reactions catalyzed by the enzymes involved in proximal pyruvate metabolism as well as the symptoms and incidences, where known, of the metabolic deficiencies characterized by their misregulation, mutation, or loss in human patients

## Cytosolic pyruvate metabolism

Cytosolic pyruvate originates from several sources (Fig. 1). In most cells, the major source of pyruvate is the last step of glycolysis, where pyruvate kinase converts phosphoenolpyruvate to pyruvate. Other significant sources include lactate via lactate dehydrogenase (LDH) and alanine via alanine aminotransferase (ALT).

### Pyruvate kinase

Pyruvate kinase (PK) catalyzes the dephosphorylation of phosphoenolpyruvate into pyruvate during the final, irreversible step of glycolysis. The breakdown of glucose via glycolysis yields two molecules of pyruvate and two net molecules of ATP. Thus, glycolysis is an important source of energy for most cells in the body. It is especially important in red blood cells which lack mitochondria and in skeletal muscle during intense periods of work, when ATP production by oxidative phosphorylation is insufficient to power muscle contraction. PK plays a prominent role here because it catalyzes one of the two energy-generating reactions in glycolysis, allowing for glycolysis to be an energy-producing pathway.

Four unique PK isozymes, PKM1, PKM2, PKL, and PKR, enable the tissue-specific regulation of PK activity [3]. The *PKLR* gene encodes both PKL and PKR transcripts through use of alternative promoters and differential splicing. PKR is found only in erythrocytes whereas PKL is expressed in the liver, kidney, and small intestines [3]. The *PKM* gene encodes the two M or muscle-type isoforms, PKM1 and PKM2, which differ by a single alternatively spliced exon [4, 5]. In addition to skeletal muscle, PKM1 is also expressed in the heart, brain, and most other tissues [6]. PKM2 is the embryonic isoform and is found in all tissues early in life. As development progresses, PKM2 is replaced with the other isoforms [7]. Elevated PKM2 is associated with cancer and will be discussed in greater detail in the “Cancer” section of this review.

Crystal structures of several rabbit PK isoforms, as well as human PKR, have been determined [8–10]. These structures show similarity between the human PKR and rabbit PKM1 isoforms, suggesting human PKM1 is similarly structured [10, 11]. The active form of PKR is a homotetramer of ~60- kDa subunits [12]. Each subunit consists of four domains: a small N-terminal domain, and domains A, B, and C. The A and C domains are involved in domain–domain interactions where each monomer is involved in head-to-head and tail-to-tail interactions with neighboring subunits [8, 10]. The phosphoenolpyruvate binding site is formed in a cleft between the A and B domains and contains the magnesium and potassium ion cofactors essential for catalysis. For the reaction to proceed, both phosphoenolpyruvate and ADP must bind within this active site. Phosphoenolpyruvate is stabilized by interactions with the magnesium and potassium cofactors. The phosphoryl group of phosphoenolpyruvate is transferred to ADP, creating ATP and enolpyruvate, which undergoes tautomerization to form pyruvate.

Domain C also contains allosteric effector binding sites. Fructose 1,6 bisphosphate is a critical positive effector for PK activity. In the absence of fructose 1,6 bisphosphate, PK exists mainly as a monomer. When fructose 1,6 bisphosphate is present, PK tetramerizes and becomes catalytically active [12]. PKR, PKL, and PKM2, but not PKM1, are regulated in this fashion [13, 14]. While PKM1 and PKM2 differ only in 21 amino acids, the region that differs is composed of two alpha-helices that are involved in subunit–subunit contacts, shifting PKM1 into a more constitutively active conformation [13, 14].

The PKL isozyme is regulated at the transcriptional and posttranscriptional level in response to various hormones. In response to glucagon, PKL is phosphorylated by PKA and inhibited, serving to inhibit glycolysis during times of glucose scarcity [7]. In contrast, insulin activates various protein phosphatases that dephosphorylate PKL, reactivating the enzyme. Glucagon and insulin also modulate *PKL* transcription [7].

Metabolic deficiencies have been reported related to the loss of the PKR isozyme [15]. This is the most common metabolic deficiency associated with glucose metabolism with an incidence of 1:20,000 [15]. Red blood cells are highly affected by the loss of PKR because they lack mitochondria and are therefore reliant upon glycolysis for ATP generation. Patients with a loss of PKR activity suffer from hemolytic anemia [16]. Low ATP levels in the red blood cell trigger hemolysis which, at high rates, leads to bilirubinemia and anemia. Severe cases of anemia may lead to death, though transfusion treatments are routinely successful. Hemolytic anemia is especially lethal in newborns because high levels of bilirubin in the brain cause tissue damage that can be lethal [17]. High bilirubin levels can be treated with a photo treatment in which light is applied to the child, breaking down the bilirubin into products which are easily eliminated from the body. Adults are not affected by bilirubinemia from red blood cell lysis because they possess a complete blood–brain barrier that prevents bilirubin access to the brain. There may be a selective advantage for heterozygotes carrying a PKR mutation as these mutations may confer some resistance to malaria [18, 19].

Numerous etiologies for PKR deficiency have been characterized, including point mutations, frameshift mutations, and large deletions within the *PKR* gene [15–17, 20–22]. Although some mutations affecting PKR will also affect PKL, liver dysfunction is very rarely observed [23]. PK deficiency, to some extent, is compensated by continuous PKL protein synthesis in the liver [16, 21–23].

### Lactate dehydrogenase

The major cytosolic fate of pyruvate produced by PK is reduction to lactate. LDH is a ubiquitously expressed enzyme that reversibly catalyzes reduction of pyruvate to L-lactate coupled with the oxidation of NADH to NAD^+^. LDH is an important enzyme in cellular metabolism, especially in skeletal muscle and cancer cells. During intense exercise, the energy requirements to support continued muscle contraction often exceed mitochondrial capacity for ATP production by oxidative phosphorylation. Furthermore, because glycolysis requires NAD^+^, ATP production by glycolysis is hindered when NAD^+^ levels diminish and NADH accumulates. LDH facilitates glycolytic ATP production by regenerating NAD^+^. With a steady supply of NAD^+^, and until acidosis becomes limiting, glycolysis can produce ATP to support work rates exceeding those that could be supported by oxidative phosphorylation alone [24, 25]. Lactate produced in muscle is transported into the blood where it circulates and is taken up by the liver. In the liver, LDH converts lactate back into pyruvate where it supports citric acid cycle flux and gluconeogenesis. This entire process is called the Cori cycle. LDH also plays an important role facilitating the Warburg Effect in cancer cells and will be discussed in the “Cancer” section.

LDH is a tetrameric complex composed of two different isoforms, termed H and M. The M isoform, or LDHA, is so named because it is the predominate isoform in the skeletal muscle, while the H isoform, or LDHB, predominates in the heart [26]. Five distinct LDH isozymes have been characterized, each differing in the ratio of H and M subunits present in the tetramer, ranging from all type H (H_4_) to all type M (M_4_) [24]. The two LDH isoforms are functionally distinct. LDHA favors the production of lactate and is not inhibited by high concentrations of pyruvate. On the other hand, LDHB favors the production of pyruvate and is inhibited by high concentrations of pyruvate [25, 27, 28]. In the liver, lactate import coupled to mitochondrial consumption of pyruvate drives the formation of pyruvate from lactate to support gluconeogenesis as part of the Cori cycle.

A third LDH isoform, termed LDHC, also forms a homo-tetrameric complex and is found only in the testes [29, 30]. Finally, the coding sequence to a fourth LDH, LDHD, has been identified. This protein sequence is highly homologous to yeast d-lactate dehydrogenases. However, these proteins, and their role in human metabolism, have remained relatively uncharacterized [31]. In mice, *Ldhd* mRNA levels are decreased upon fasting, and return to basal levels upon refeeding [32]. Additionally, *Ldhd* mRNA levels are increased in mouse models of type 2 diabetes [33].

LDHA deficiency is characterized by the complete absence of the M isoform [34, 35]. The symptoms of LDHA deficiency include myoglobinuria resulting from muscle degeneration, low endurance, elevated blood pyruvate levels, and, in some cases, skin disorders. Interestingly, this deficiency is not lethal. It seems that, to some extent, the H isoform can compensate for the loss of the M isoform in most tissues. Conversely, LDHB deficiency, characterized by complete loss of the H isoform, is largely asymptomatic [34]. Additional mutations have also been identified in both *LDHA* and *LDHB*, though symptoms are relatively mild and not life-threatening [36].

### Alanine aminotransferase

Pyruvate can be generated through the catabolism of various amino acids, including alanine, serine, and threonine. Alanine is worthy of special consideration because it is one of the major gluconeogenic precursors [37]. ALT, also frequently referred to as glutamic pyruvate transaminase or GPT, catalyzes the reversible transamination of alanine and α-ketoglutarate to glutamate and pyruvate. These four intermediates function as important links between carbohydrate and amino acid metabolism.

Pyruvate and alanine are the central substrates in the alanine cycle, a recycling and scavenging pathway linking muscle and liver metabolism [38]. In muscle, pyruvate is transaminated into alanine and exported from the cell. The liver recovers the alanine and deaminates it back to pyruvate, which supports citric acid cycle flux and the multiple pathways intersecting it. The alanine cycle is quite similar to, and often occurs in parallel with, the Cori cycle. However, the alanine cycle is less efficient than the Cori cycle because glutamate is deaminated by glutamate dehydrogenase, creating α-ketoglutarate. This reaction produces ammonia, which must be detoxified by the urea cycle [39].

Two isoforms of ALT have been identified. ALT1 (~54 kDa) is localized to the cytosol. ALT2 (~57 kDa) is found in the mitochondrial matrix. Both isoforms display different, but overlapping, tissue expression profiles. ALT1 is more strongly expressed in brown and white adipose tissue, intestine, and liver. ALT2 is strongly expressed in the muscle and the brain. Interestingly ALT1 and ALT2 expression profiles vary greatly between different species. In some species, like rats and mice, both ALT1 and ALT2 are highly expressed in the liver. In contrast, ALT1 is the predominate isoform in humans, with little or no expression of ALT2 in the liver [40, 41]. A splice variant of ALT2 termed ALT2-2, has been discovered, which displays no aminotransferase activity and lacks the first ~100 amino acids found in ALT2 [42]. The physiological significance of this isoform is currently unknown.

Historically, serum ALT activity has been an important biomarker signaling liver damage. Normally an intracellular protein, elevated levels of ALT activity in the blood has been taken to indicate liver tissue damage during which the cellular components leak into the circulating blood supply [43]. More recently, isoform specific ALT assays have been developed that are able to differentiate between injuries that increase ALT1 or ALT2 levels in the blood. For example, in response to liver damage, the proportion of ALT1 specific activity increased in the serum. In response to muscle damage the proportion of ALT2 specific activity increases [40].

ALT is regulated at multiple levels. Fasting and refeeding experiments have shown that both *ALT1* and *ALT2* mRNA levels increase during fasting and return to baseline upon refeeding [32]. This is to be expected as, during prolonged fasting, amino acids are used as a fuel source. Since alanine can be converted directly into pyruvate, it serves as a key gluconeogenic precursor. In non-hepatic tissues *ALT2*, but not *ALT1*, expression is regulated by androgens [44]. Finally, multiple acetylation sites on ALT2 have been identified, though the functional significance has yet to be determined [45, 46]. In vitro experiments have shown that ALT1 can be inhibited by spontaneous glycation [47].

To date, no severe metabolic defect has been conclusively associated with ALT deficiency. Multiple ALT null patients have been described [48–51]; however, ALT deficiency may predispose, or be secondary to, the disorders described in these reports. In fact, far more concern has been expressed in the potential misdiagnoses that could result from the artificially reduced serum ALT activity levels in null patients rather than any deleterious phenotype [51].

## Mitochondrial pyruvate metabolism

Pyruvate kinase, lactate dehydrogenase, and alanine aminotransferase are the major sources of cytosolic pyruvate. Once produced in the cytoplasm, most pyruvate is ultimately destined for the mitochondrial matrix. In the matrix, carbon from pyruvate drives citric acid cycle flux thereby supporting ATP production by oxidative phosphorylation and multiple biosynthetic pathways (Fig. 2).

### Mitochondrial pyruvate carrier

The mitochondrial pyruvate carrier transports pyruvate from the mitochondrial intermembrane space to the mitochondrial matrix. Pyruvate and other small molecules freely diffuse from the cytoplasm to the intermembrane space through porins. However, the inner mitochondrial membrane is impermeable to charged molecules, which enables it to sustain the proton gradient necessary for oxidative phosphorylation. To transit the inner mitochondrial membrane and reach the matrix, pyruvate requires a specific carrier, the MPC. Thus, the MPC effectively links cytosolic pyruvate metabolism with the citric acid cycle. While the existence of the biochemically inhibitable MPC activity has been known for several decades [52–55], the molecular identify of the MPC was only recently discovered [1, 2].

In humans, the MPC is formed by two paralogous subunits, MPC1 and MPC2, in a currently unknown stoichiometry [1, 2]. Little is also known about the physiological regulation of the MPC. It has been reported that MPC activity is increased in response to glucagon and decreased in response to insulin [56, 57]. Large-scale transcriptome and mitochondrial proteome studies have revealed that molecular regulation does occur. For example, fasting and refeeding studies in mice have shown that *MPC2* transcript levels increase approximately 1.5-fold under fasting conditions compared to baseline and refed conditions [32]. Furthermore, in mice, acetylation of MPC2 on K19 and K26 has been observed [45]. Finally, hydroxylation of MPC2 on P92 has also been observed [46]. However, the physiological relevance of these posttranslational modifications is currently unknown. Regulation of MPC1 was not reported but cannot be excluded because targeted studies were not performed.

The *MPC1* and *MPC2* paralogs almost certainly arose from an ancient, though as of yet unidentified, gene duplication event. Both proteins are predicted to contain two to three transmembrane domains [1, 2]. Interestingly, they do not contain any sequence homology to other known mitochondrial carrier proteins, such as the phosphate carrier PiC or the adenosine nucleotide transporter ANT, as was previously proposed [58]. They have recently been proposed to belong to the PQ-loop/MtN3/MPC superfamily [59]. PQ-loop family members perform diverse functions throughout the cell and are located in a variety of organelles, including the mitochondria, ER, Golgi, and, in plants, chloroplasts [60]. Characteristics typical of the PQ-loop family include seven transmembrane domains and two conserved proline glutamine motifs. MPC1 and MPC2 are only half the size of other PQ-loop family members, each containing potentially three of the typical seven transmembrane domains [60]. Thus, MPC1 and MPC2 may be homologous to the N-terminal or C-terminal halves of the other PQ-loop proteins. Inclusion into this family assumes that MPC1 and MPC2 homo- or heterodimerize and fold into a structure reminiscent of full-length PQ-loop proteins. Further work is required to determine whether MPC1 and MPC2 are true members of the PQ-loop family.

Mitochondrial pyruvate uptake has been proposed to be coupled with the electrochemical gradient, occurring with the symport of one proton, or alternatively, exchange with one hydroxide ion [55, 61]. Pyruvate is the most important, but not sole, substrate of the MPC. Compounds such as dichloroacetate and other small halogenated monocarboxylates can be transported by the MPC, and this transport can be inhibited [53, 55]. Several compounds have been shown to inhibit MPC activity, including α-cyano-4-hydroxy cinnamate, UK-5099, and several thiazolidinediones compounds [53, 55, 62, 63]. Both UK-5099 and α-cyano-4-hydroxy cinnamate have been reported to also inhibit the monocarboxylate transporters found at the plasma membrane and therefore potentially cellular pyruvate uptake. However, α-cyano-4-hydroxy cinnamate is approximately 30-fold and UK-5099 is approximately 300-fold more potent at inhibiting the MPC compared to the monocarboxlate transporters [53, 64–67].

Mutations in *MPC1* have been linked in three families to pyruvate transport deficiency [1, 68]. The first case was reported in 2003 and characterized a patient with a homozygous R97W mutation in *MPC1* [68]. The patient displayed severe developmental delay and died at age 19 months. The patient suffered from lactic acidosis that was not responsive to bicarbonate treatment. PDH activity was normal. A second *MPC1* mutation, encoding an L79H change, has also been identified, but no clinical description has been reported except that the phenotype is less severe than the R97W mutation [1]. The incidence of functional *MPC1* and *MPC2* mutations is unknown and awaits further study.

Several additional key questions regarding the MPC are still unknown with many of them centered on the regulation of the MPC. For example, are *MPC1* and *MPC2* transcriptionally regulated in response to normal physiological stimuli leading to changes in protein abundance? Is the MPC activity regulated by posttranslational modification of the MPC proteins? What is the structure of MPC complex and how do the R97W and L79H mutations impair transport? Is the MPC activity pathologically misregulated in diseases featuring aberrant pyruvate metabolism? Is the MPC a viable therapeutic drug target for these disorders? Given the critical node the MPC inhabits within cellular metabolism, changes in MPC function may play a prominent role in metabolic disease.

### Pyruvate dehydrogenase

After passage through the MPC, pyruvate has several potential fates within the mitochondrial matrix. However, the majority is oxidized to carbon dioxide by the citric acid cycle to ultimately support the generation of ATP by oxidative phosphorylation. Over a sequence of reactions, the pyruvate dehydrogenase complex (PDH) irreversibly converts pyruvate and NAD^+^ into acetyl-CoA, NADH, and carbon dioxide. The acetyl-CoA enters the citric acid cycle. Acetyl-CoA may also be used to drive multiple anabolic processes, including lipogenesis, the formation of cholesterol, and the generation of acetylcholine, a key neurotransmitter (Fig. 2). NADH and FADH_2_ are produced from the reactions of the citric acid cycle and are utilized to generate the proton gradient necessary for oxidative phosphorylation. Thus, PDH serves to bridge glycolytic metabolism in the cytosol with the citric acid cycle and oxidative phosphorylation [69, 70].

Given the central role PDH plays in cellular metabolism, its activity must be finely regulated to maintain cellular energy homeostasis as well as supply necessary carbon to the biosynthetic pathways intersecting the citric acid cycle (Fig. 2). The activity of the PDH complex is fine-tuned by the energy state of the cell. High amounts of ATP, NADH, and acetyl-CoA all inhibit the complex [69, 71]. The genes of the PDH complex are also regulated transcriptionally. Under times of energetic stress, such as fasting, transcripts for PDH-complex proteins are downregulated. Transcript levels return to baseline levels upon refeeding [32]. Rapid regulation of the PDH activity is achieved by phosphorylation and dephosphorylation, functions performed by the pyruvate dehydrogenase kinases (PDK) and the pyruvate dehydrogenase phosphatases (PDP). Both PDK and PDP, and the roles they play in the regulation of PDH, will be discussed in greater detail following this section.

PDH is a massive protein complex weighing in at ~9.5 MDa and is composed of four protein sub-complexes: pyruvate dehydrogenase (E1), dihydrolipoamide acetyltransferase (E2), dihydrolipoamide dehydrogenase (E3), and E3 binding protein (E3BP, also known as PDH Protein X) [72]. The central core structure is comprised of E2 and E3BP [73–75], which in turn recruit E1 and E3 [76].

E1 is a heterotetrameric complex containing two copies each of the proteins E1α and E1β [77]. Some 20–30 E1 complexes associate with the E2/E3BP core complex through interactions between E1 and E2 [72]. The E1 active site is a deep cleft formed at the interface between the α and β subunits and contains a thiamine pyrophosphate cofactor and a magnesium ion. The PDH reaction begins here in which the oxidative decarboxylation of pyruvate is coupled with the reductive acetylation of the lipoamide cofactor. This acetyl group is subsequently transferred from thiamine pyrophosphate to a lipoate moiety covalently bound to E2.

E2 catalyzes the transfer of the acetyl group from the lipoate moiety to CoA, forming acetyl-CoA and dihydrolipoate. Between 40 and 42 E2 subunits are found per PDH complex. Structurally, E2 is composed of four domains, each connected by a flexible linker. Starting at the C-terminus, these domains include an inner domain, a subunit binding domain, and two lipoyl domains, named lipoyl domain 1 (L1) and lipoyl domain 2 (L2) [74, 75]. The inner domain mediates formation of the core complex with E3BP and contains the acetyltransferase catalytic activity. The subunit binding domain binds and recruits E1 to the core complex. The lipoyl domains contain covalently bound lipoate moieties. These lipoate moieties are sequentially transferred between the E1, E2, and E3 active sites via a so-called ‘swinging arm’ mechanism. More recently, an alternative role for E2 has been discovered: E2 has been shown to localize to the nucleus and act in signal transduction pathways [78].

E3 catalyzes the regeneration of the lipoate group from dihydrolipoate. The oxidation of dihydrolipoate is initially performed by reduction of a bound FAD to FADH_2_. However, FADH_2_ is in turn re-oxidized by NAD^+^, forming NADH and regenerating FAD [79]. Once regenerated, FAD and the lipoate group can participate in the next reaction cycle. Structurally, E3 is a homodimer with 6–12 E3 complexes associating with the core PDH complex through interactions between it and the subunit binding domain of E3BP. Each E3 dimer can interact with two E3BP proteins at the subunit binding domain, creating crosslinks between E3BP proteins [76].

E3 is associated not only with the PDH complex but also with the α-ketoglutarate dehydrogenase complex and the branched chain amino acid dehydrogenase complex, where it performs a similar catalytic function. However, E3 binds PDH much more strongly compared to the core complex of the branch chain amino acid dehydrogenase complex [80, 81]. This difference is attributed to the presence of an arginine residue in the E3 binding protein of the branched chain amino acid dehydrogenase complex, whereas an asparagine is in the PDH complex. It is thought that the larger arginine residue causes a steric clash, reducing the binding affinity [80, 81].

E3BP is the final component of the PDH complex and is a structural protein with no enzymatic function. E3BP is organized in a similar fashion as E2, and contains an inner domain, a subunit binding domain, and a single lipoyl domain, lipoyl domain 3 (L3) [76, 82]. Between 18 and 20 E3BP are found in each PDH core complex. E3BP is critical for the proper formation of the central core structure and the recruitment of E3 to the complex [73, 74].

Given the critical role PDH plays in cellular energy metabolism and biosynthetic pathways, multiple levels of regulation are applied so that the demands of the cell are balanced. A critical regulatory mechanism is the reversible phosphorylation of three serine residues, Ser-264 (site #1), Ser-271 (site #2), and Ser-203 (site #3), on the E1α subunit [83], a role performed by PDK. Four isoforms of PDK have been characterized, though these proteins will be explored in greater depth below [84, 85]. Phosphorylation of any site is sufficient to ablate enzymatic activity. Site 1 is the most frequent target [86, 87]. These serine residues are located in loops which, upon phosphorylation, lose the ability to bind and recruit the lipoyl domains to the activity site of E1 [83]. Counteracting the PDKs are the PDPs, which dephosphorylate E1α, restoring PDH activity. Two PDP isoforms have been characterized and will be explored in greater detail below [88].

### Pyruvate dehydrogenase complex deficiency

Pyruvate dehydrogenase complex deficiency is defined by reduced PDH activity in patient cells [89]. To date, mutations in all four PDH subunits have been described that cause PDH deficiency. The severity of the deficiency varies widely depending upon the mutation and the subunit affected. Deficiencies in specific subunits will be discussed below. For a more in-depth discussion of PDH deficiency, the reader is recommended to two excellent recent reviews [89, 90]. Symptoms of PDH deficiency include lactic acidosis, elevated pyruvate levels, and ataxia. In longer-lived patients, symptoms include developmental delay, psychomotor retardation, and decreased cognitive capacity [89–91]. These symptoms demonstrate that the nervous system, due to its reliance upon carbohydrate metabolism, is especially sensitive to perturbations in PDH activity.

Treatment of PDH deficiency varies greatly, due in part to the multiple etiologies of this disease [92]. In many cases, treatment with bicarbonate is initiated to counteract the lactic acidosis. Treatment with thiamine and dichloroacetate has been successful in some, though not all, cases. Dichloroacetate is a well-known inhibitor of PDK and therefore causes increased PDH activity. Thiamine is a precursor to the thiamine pyrophosphate cofactor present in the active site of E1. Thiamine supplementation increases the fraction of PDH complexes which have adequate thiamine pyrophosphate. Additionally, a ketogenic diet, which is a diet high in lipid calories and low in carbohydrate calories, is often prescribed. This decreases the overall reliance of acetyl-CoA generation on mitochondrial pyruvate and PDH. Instead, alternative metabolic pathways, such as β-oxidation of lipids and use of ketone bodies, are used for ATP production. However, the overall prognosis is poor and most patients die at a young age even with treatment [89–92].

Mutations present in the X-linked *E1α* subunit are the most common cause of PDH deficiency [89, 90]. Missense and frameshift mutations have been identified that affect the ability of E1α to bind the thiamine pyrophosphate cofactor, to properly assemble into the heterotetramer, and to be properly targeted and transported into the mitochondria. Defects in *E1β* are rarer and primarily characterized by point mutations that disrupt the formation of the E1 heterotetramer and E1 catalytic activity [93].

Defects in E2 are extremely rare, with less than ten cases having been reported in the literature [91, 94, 95]. The cause of E2 deficiency includes point mutations and mRNA mis-splice events. Patients with this defect do not display the lactic acidosis typical of PDH deficiency, and overall PDH activity is decreased only ~30–50 %. Afflicted patients exhibit developmental delay and psychomotor motor retardation but can survive into adulthood when placed on a ketogenic diet [91].

E3 deficient patients present symptoms similar to those above but suffer additional complications because E3 is involved in multiple dehydrogenase complexes, such as the branched chain amino acid degradation pathway. This results in elevated levels of valine, leucine, and isoleucine [79–81, 92]. Point mutations, frameshift mutations, and mRNA processing defects have all been characterized to cause E3 deficiency. Point mutations, by far the most common defect, affect subunit organization, FAD binding, and NAD^+^ binding [79, 89].

Finally, *E3BP* mutations have also been reported, including the complete loss of E3BP due to a nonsense mutation [89, 95–97]. Patients were reported to have developmental delay, elevated pyruvate, lactate, and alanine levels, as well as reduced PDH activity (~27–33 % control). No E3BP protein was detected in patient samples [96]. It is not currently known how the loss of E3BP greatly affects PDH activity. Multiple hypotheses can be generated. First, loss of E3BP may cause the core structure of PDH to be lost, compromising the precise channeling of substrate from one enzyme to the next. This would decrease the overall efficiency of the reaction and may explain the decreased PDH activity. Alternatively, the central core could still be formed, composed solely of E2 subunits, as is seen in purified E2 protein preparations. Thus, the main effect on PDH activity would be diminished E3 recruitment to the core complex.

### Pyruvate dehydrogenase kinase

The rapid downregulation of PDH activity is achieved by phosphorylation of the E1α subunit; a task performed by PDK. Phosphorylation of PDH decreases its activity, reducing flux through PDH and downstream metabolic pathways. This results in the overall conservation or redirection of mitochondrial pyruvate to other metabolic fates. This is important, for example, during fasting, in which pyruvate is utilized to produce glucose via gluconeogenesis to maintain blood sugar levels.

Four PDK isoforms have been characterized, termed PDK1–PDK4 [84, 85]. These isoforms vary slightly in size. PDK1 is the largest at ~48 kDa; the remaining three isoforms are ~45 kDa. Each isoform displays unique, but overlapping tissue expression profiles. For example, all four isoforms are present in the heart and skeletal muscle, though PDK2 and PKD4 predominate. PDK3 is found only in heart and skeletal muscle. PDK2 is highly expressed in heart, skeletal muscle, and the liver. PKD4 is highly expressed in kidneys, brain, and liver [84, 85, 98–101]. Active PDK is a dimer, and can be either a homo- or heterodimer, depending on whether the tissue under examination expresses more than one PDK isoform [102].

Recruitment of PDK to the PDH complex is facilitated by binding of PDK to either the inner lipoyl domain 2 [103] or the outer lipoyl domain 1 of E2 [104]. Only 1–2 copies of PDK are associated with each PDH complex [72]. Therefore, for fully inactive PDH, PDK must move across the entire surface of PDH. A hand-over-hand model has been proposed. At any given time, one of the PDK subunits is bound to an inner lipoyl domain 2. The free subunit is then able to swing around and bind with other nearby lipoyl domain 2s. In this fashion, PDK can move across PDH without dissociating from the complex [100, 105].

Structurally, each PDK polypeptide chain is composed of two domains, termed the C-terminal domain and the N-terminal domain. The C-terminal domain is involved in mediating the dimerization of PDK subunits through extensive B-sheet interactions [101]. The active site is a cleft formed at the interface between the two domains. Each of the four isoforms is kinetically distinct, with slightly different specific activities towards the E1 phosphorylation sites, binding affinities for the lipoyl domain 2, and stimulation upon lipoyl domain 2 binding. For example, all four isoforms are able to phosphorylate sites 1 and 2, but only PDK1 can phosphorylate site 3 [86]. PDK3 has the highest specific activity toward site 2, while PDK2 has the highest for site 1 [86, 87, 106]. The binding affinity of the PDK isoforms for lipoyl domain 2 varies. PDK3 has been shown to have the strongest affinity for lipoyl domain 2 while PDK4 displays the weakest affinity [103, 104]. Finally, in some cases, the specific activity and binding affinity of PDK for lipoyl domain 2 is modulated by the redox state of the lipoyl group. The apparent activity of PDK increases when the lipoyl moiety on lipoyl domain 2 is in a reduced or acetylated state (active). Conversely, specific activity is decreased when the lipoyl moiety is oxidized (inactive or resting state) [104].

PDK occupies an important role in controlling metabolic pyruvate flux, and, as such, is highly regulated. PDK isoforms are transcriptionally regulated, and this regulation is tissue-dependent. For example, during fasting conditions, *PDK2* and *PDK4* expression is increased in the liver, consistent with conditions of decreased carbohydrate oxidation [107]. Posttranslational modifications have also been identified. PDK1 has recently been shown to be phosphorylated by the receptor tyrosine kinase fibroblast growth factor receptor 1, which increases the activity of PDK1 by several-fold [108].

Unlike the other enzymes discussed in this review, no metabolic deficiencies have been identified with PDK as the root cause. This may indicate that other isoforms are able to compensate for the loss of one.

### Pyruvate dehydrogenase phosphatase 

Pyruvate dehydrogenase phosphatase acts in opposition to PDK by removing the phosphorylation marks on PDH E1α, thereby reactivating the PDH complex. Reactivation of the PDH complex increases acetyl-CoA flux into the citric acid cycle to support oxidative phosphorylation or biosynthesis.

Two PDP isoforms, PDP1 and PDP2, are found in humans [88]. Each isoform displays distinct but overlapping tissue expression profiles. PDP1 is highly expressed in the brain, heart, skeletal muscle, and testis, while PDP2 is highly expressed in the liver and adipose tissue [109]. Structurally, PDP1 is a heterodimer composed of a catalytic subunit of ~ 52 kDa and a regulatory subunit of ~ 97 kDa. Only a catalytic subunit for PDP2 has been identified. A regulatory subunit is hypothesized to exist but has yet to be found [110]. The catalytic subunit of both PDP isoforms is related to the protein phosphatase 2C family of serine phosphatases [88, 111]. Two magnesium ions cofactors are required for activity.

PDP1 and PDP2 are themselves regulated. In response to fasting, *PDP1* and *PDP2* mRNA and protein levels decrease, though the differences are isoform- and tissue-specific to some extent. Decreases in PDP abundance and activity could function to reduce PDH activity for channeling of pyruvate carbon into gluconeogenesis during fasting. Levels return to basal conditions upon refeeding [109]. Additionally, PDP1 and PDP2 are phosphorylated by PKCδ which increases PDP activity, and, subsequently, PDH activity [112]. Activity of PDP1, but not PDP2, is increased in response to calcium. Calcium in skeletal muscle, where PDP1 is highly expressed, stimulates muscle to contract and do work. Therefore, calcium, acting through PDP1, signals to increase the activity of PDH. This facilitates increased mitochondrial ATP production to support the energetic demands of muscle contraction. PDP2 activity is increased in the presence of spermine, a naturally produced polyamine, though the biological significance of this interaction is still unclear [88].

Several reports have highlighted PDP deficiencies in human patients. In 2005, a case study with two patients described a frameshift mutation causing the in-frame deletion of leucine-213 [113]. Patient symptoms included elevated lactate levels, hypotonia, feeding difficulties, and exercise intolerance coupled with slight developmental delay. According to structural information for the catalytic domain of PDP1, it has been proposed that removal of Leu-213 disrupts the position of Asp-220 [111, 113]. Asp-220 is part of a hydrogen bond network critical for the proper structure of the active site. This frameshift mutation may also have caused protein instability as PDP1 levels were ~20 % of wild-type [113]. At the time of the report, both patients were alive and being treated with a ketogenic diet.

A second report published in 2009 described a mutation in *PDP1* creating a premature stop codon (E93X), generating a null mutation with no detectable PDP1 protein [114]. This patient presented symptoms including lactic acidosis and elevated alanine and proline levels. Bicarbonate treatment was well-received and maintained; however, at 6 months of age, the patient died of acute respiratory distress. Lysates from patient fibroblasts showed greatly reduced PDH activity that could be corrected by addition of recombinant PDP1 protein or dichloroacetate. This study also showed that PDP2 can, to some extent, compensate for loss of PDP1.

### Pyruvate carboxylase

As an alternative to decarboxylation by PDH, the second major fate of mitochondrial pyruvate is the irreversible, ATP-dependent carboxylation of pyruvate to oxaloacetate by pyruvate carboxylase (PC) [115, 116]. Oxaloacetate is a critical intermediate in metabolism, linking carbohydrate, lipid, amino acid, and nucleotide metabolism (Fig. 2) [117–119].

The utility of mitochondrial pyruvate is not limited to the production of ATP but also includes providing carbon to several major biosynthetic pathways intersecting the citric acid cycle (Fig. 2). Many citric acid cycle intermediates are important for the biogenesis of the non-essential amino acids. For example, α-ketoglutarate is a key intermediate for the biogenesis of glutamine, glutamate, arginine, and proline, while oxaloacetate is used to generate aspartate and asparagine [119]. Oxaloacetate and citrate also support the major biosynthetic pathways of gluconeogenesis and lipogenesis, respectively. Heme, a key biological molecule important for the transport of oxygen throughout the body, is produced from succinyl-CoA [120]. However, the pool of citric acid cycle carbon is limited. Any intermediates removed for biosynthetic purposes must be replenished in order to maintain citric acid cycle carbon flux. Reactions that replenish citric acid cycle intermediates are termed anaplerotic [119]. Oxaloacetate generated by PC fulfills a critical role anaplerotically replenishing the citric acid cycle by serving as an acceptor for acetyl-CoA produced by PDH. Another key anaplerotic pathway is the catabolism of glutamine and glutamate to α-ketoglutarate, which is especially vital for the growth of many cancer cells [121].

In humans, a single PC isoform is expressed and found only in the mitochondrial matrix [122, 123]. Structurally, PC is a homotetramer, arranged as a dimer of dimers, with each subunit approximately 120 kDa in size. The quaternary structure of PC is necessary as monomeric PC has no activity. PC contains four distinct domains which include, starting at the N-terminus, a biotin carboxylase domain, the first half of an allosteric regulatory domain, a carboxyl transferase domain, the second half of the allosteric regulatory domain, and a biotin carboxyl carrier protein domain [117, 124, 125]. The reaction begins at the active site of the biotin carboxylase domain where a carboxy-biotin intermediate is produced from ATP, bicarbonate, and biotin. The carboxybiotin group is transferred to a neighboring carboxyl transferase domain [117, 118, 125]. There, the carboxyl group is transferred from carboxybiotin to pyruvate, generating oxaloacetate and regenerating the biotin cofactor.

Given PC’s importance in cellular metabolism, its activity is tightly regulated. *PC* expression is nearly ubiquitous throughout the body, though higher expression levels are found in certain tissues, such as the liver, kidney, adipose tissue, and the heart [116]. PC specific activity is positively regulated by acetyl-CoA [118]. High acetyl-CoA levels indicate that either (1) cellular energy demand is being met and pyruvate should utilized for gluconeogenesis or (2) that there is inadequate oxaloacetate to accept the acetyl-CoA being produced by PDH during energetic stress. Under the first condition, PC plays the critical role of channeling pyruvate carbon towards gluconeogenesis. Under the second, PC provides a critical anaplerotic shunt to maintain citric acid cycle carbon flux. The combined regulation of PC and PDH determines where overall carbon flux is channeled.

In accord with PC’s critical role in gluconeogenesis, it is regulated by fasting and refeeding. In response to insulin, PC activity is downregulated, diminishing gluconeogenic carbon flux during times of high glucose levels. In response to fasting, *PC* mRNA levels increase and support increased gluconeogenesis [45, 46, 118, 126]. More recently, mitochondrial proteome studies have identified a hydroxylation site and multiple acetylation sites on PC [45, 46]. However, the physiological significance of these modifications, if any, is not yet known.

Deficiencies in PC vary in type and severity and are generally classified into three groups [127–129]. Type A, or the North American type, is associated with decreased, but not completely absent, PC activity. These patients exhibit elevated alanine and proline levels and episodic lactic acidosis, and suffer from some form of developmental delay. However, with treatment, patients can survive for several years. Type B, or French Type, PC deficiency is generally characterized by nearly absent PC activity, and in many cases absent PC protein [92]. These patients suffer from severe lactic acidosis and neurological issues, and generally do not survive longer than several months [127]. Finally, Type C is a relatively mild form of PC deficiency. These patients suffer episodes of lactic acidosis but do not display the neurological issues shown by Type A or Type B patients [127, 129, 130]. These cases are rare and the molecular basis is unknown [131]. The molecular bases of Type A and Type B PC deficiency are varied, with some overlap. Type A patients tend to harbor missense mutations that reduce PC activity. Type B patients also harbor missense mutations and display more severe defects that ablate mature protein expression, such as mis-splice events and truncations [127].

## Major diseases characterized by aberant pyruvate metabolism

Pyruvate occupies a critical node in central carbon metabolism and, as discussed above, altered pyruvate metabolism can cause disease. Aberrant pyruvate metabolism plays an especially prominent role in cancer, neurodegeneration, heart failure, and other conditions that will be discussed below.

### Cancer

**Fig. 3:**
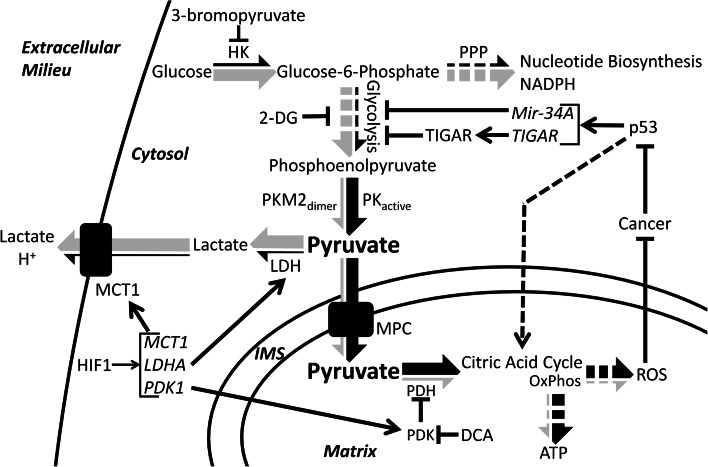
Pyruvate dysmetabolism in cancer. Pyruvate metabolism and carbon flux is altered in many cancer cells. *Arrows* show the relative carbon flux through different metabolic pathways in cancer (*gray portion* of the *large arrows*) and in normal cells (*black portion* of the *large arrows*). Generally, cancer cells upregulate glycolysis and the pentose phosphate pathway and downregulate the citric acid cycle and oxidative phosphorylation. Modulators of cancer metabolism include: HIF1, which upregulates transcription of *MCT1*, *LDHA*, and *PDK1*; p53, which downregulates glycolysis by transcriptional induction of *Mir*-*34A* and *TIGAR*, upregulates oxidative phosphorylation (OxPhos), and is typically silenced in cancer; and dimeric PKM2, which impairs pyruvate production and therefore OxPhos and also causes accumulation of glycolytic intermediates and increased biosynthetic carbon flux through the pentose phosphate pathway (*PPP*). Finally, several therapeutics and their effects are shown: 2-deoxyglucose (*2-DG*) and 3-bromopyruvate directly inhibit glycolytic enzymes thereby decreasing aerobic glycolysis. DCA inhibits PDK thereby activating PDH and increasing citric acid cycle flux and oxidative phosphorylation. *HK* hexokinase, *PPP* pentose phosphate pathway, *DCA* dichloroacetate, *2-DG* 2-deoxyglucose, *TIGAR* tp53 induced glycolysis and apoptosis regulator

Many cancer cells are, in part, defined by a metabolic switch termed the Warburg Effect, in which glycolytic carbon flux is highly upregulated while oxidative phosphorylation is significantly downregulated (Fig. 3) [132–134]. The factors involved in this metabolic switch are many, and vary according to cancer type. Within the context of this review, major factors such as PKM2, HIF1, and p53 will be discussed in terms of their relationships to pyruvate metabolism. Potential therapies will be briefly discussed. For a more general treatment of these proteins, the reader is suggested to read the many excellent recent reviews, on PKM2 [135, 136], HIF1 [137], and p53 [138]. It should also be noted that some cancers do not display the Warburg Effect, and many only partial aspects depending upon stage or location. The foregoing are features of many but not all cancers.

A direct consequence of the Warburg Effect is the highly elevated production of lactate as the primary metabolic end product. Furthermore, lactate itself is used to further advantage by cancer cells. First, the conversion of pyruvate to lactate regenerates the NAD^+^ cofactor necessary for the continuation of glycolysis. Second, export of lactate out of the cell, which is facilitated by the monocarboxylate transporters, is proton-linked, contributing to the acidification of the extracellular environment surrounding the cancer cells [132]. Acidification of the extracellular environment provides a measure of protection from the immune system [132]. For example, an acidic environment and high lactate concentrations impair the ability of cytotoxic T lymphocytes, white blood cells which destroy infected or damaged cells, to proliferate, produce cytokines, and mediate the destruction of cancer cells [139, 140]. Furthermore, lactic acid appears to influence the activity of matrix-associated metalloproteinases, which breakdown the extracellular matrix adjacent to the tumor, aiding in proliferation and metastasis [141–143]. Finally, lactate can be utilized as a fuel source by cancer cells located at the surface of the tumor, where, after conversion back to pyruvate, oxygen levels are sufficient to support oxidative phosphorylation. Glucose is thereby conserved for the cancer cells buried inside tumor [144]. Thus, cancer cells derive immense benefit from diverting pyruvate from its normal cellular fate and converting it into lactate.

A key factor influencing the transition away from pyruvate oxidation and towards the Warburg Effect is the transcription factor hypoxia inducible factor 1, or HIF1. HIF1 is an important regulator of pyruvate metabolism and is frequently overexpressed in many cancers [145]. HIF1 is a heterodimer composed of HIF1α, a basic helix loop helix transcription factor, and HIF1β (also known as aryl hydrocarbon receptor nuclear translocator, or ARNT). Under normoxic conditions, HIF1α is hydroxylated at either Pro402 or Pro564, or both, which targets the protein for proteosomal degradation [146–148]. However, under hypoxic conditions, hydroxylation does not occur, stabilizing HIF1α and targeting it for translocation into the nucleus [141, 145]. HIF1β, on the other hand, is a constitutively expressed transcription factor which is involved in several other signaling pathways, most notably the detoxification of polyaromatic hydrocarbons [149]. Several hundred genes are transcriptionally regulated by HIF1. In regards to pyruvate metabolism, HIF1 regulates factors involved in glycolysis as well as PDK1, MCT4, and LDHA [150, 151]. PDK1 inhibits the PDH complex and has been discussed in greater detail within this review. MCT4, or monocarboxylate transporter 4, is a member of a family of membrane proteins which mediate the translocation of small monocarboxylates, like pyruvate and lactate, across the plasma membrane. MCT4 is frequently upregulated in cancer cells and functions in the export of lactate plus a proton from the cytoplasm into the extracellular environment [152]. Finally, LDHA catalyzes the formation of lactate from pyruvate, which can then be used for myriad purposes as discussed above. Indeed, the upregulation and overexpression of LDHA is an important part of cancer metabolism, as knockdown affects cancer progression, proliferation, and survival [153]. Furthermore, HIF1 upregulates MIX Interactor 1, a transcription factor that represses cMYC, leading to a decrease in mitochondria biogenesis and mass, further decreasing pyruvate oxidation [151, 154]. Due to the important role HIF1 performs in mediating the metabolism and survival of cancer cells, it has become an important therapeutic target. Multiple aspects of HIF1 biology have been targeted, including regulation of protein abundance, protein stability, and transcript abundance, among others. Reduction of HIF1 activity is correlated with reduced tumor growth and reduced metastatic ability [145, 155].

Cancer cells further modulate pyruvate metabolism through the downregulation of important cellular regulators, such as p53. P53 is a master regulator of the cell well known for the role it plays in cell cycle control, apoptosis, DNA damage and repair, and metabolism [138, 156, 157]. p53 functions as a tumor suppressor by halting cell cycle progression and by activating apoptosis in response to cellular damage. More recently, however, p53 has been shown to function as a tumor suppressor by directly modulating glucose metabolism through the transcription of mir-34A, a micro-RNA [157]. After proper processing, mir-34A is loaded into the RNA-induced silencing complex and mediates the silencing of many glycolytic enzyme transcripts, resulting in an overall decrease in glycolysis. Furthermore, p53 upregulates the transcription of tp53-induced glycolysis and apoptosis regulator, or TIGAR, which can function to metabolize fructose 2,6 bisphosphate [158], thereby suppressing the activity of phosphofructokinase 1 and glycolysis in general [138, 156]. Thus, p53 functions to regulate glycolysis, and therefore pyruvate formation and oxidation, in normal cells. In cancer cells with silenced p53, this level of control is lost, contributing to the increase in glycolysis as well as pyruvate and lactate formation. It is likely, however, given the myriad roles p53 plays, that additional functions and pathways will be elucidated linking p53 activity to cellular metabolism. It is not surprising then that p53 is frequently silenced or mutated in cancer cells [138].

Finally, cancer cells directly alter pyruvate metabolism by shifting the expression pattern of glycolytic enzymes, specifically that of pyruvate kinase, in favor of the M2 (PKM2) isoform [6, 108]. Unlike the other PK isoforms, which form stable tetramers [159], PKM2 has been shown to associate as either a tetramer or a dimer. Homotetrameric PKM2 is highly active and efficiently catalyzes the formation of pyruvate from phosphoenolpyruvate. In contrast, dimeric PKM2 is essentially inactive [135, 160]. Interconversion of PKM2 between the dimer and tetramer forms is quite dynamic [135, 136], and can be modulated by various posttranslational modifications as well as several allosteric effectors and binding partners [159, 160]. Cancer cells take advantage of the dynamic nature of PKM2 to modulate glucose-derived carbon flux. Dimeric (inactive) PKM2 creates a constriction through glycolysis at the terminal reaction resulting in the accumulation of glycolytic intermediates [6, 159], which are channeled into other pathways, such as the pentose phosphate pathway, which is an anabolic pathway which generates precursors for nucleotide and aromatic amino acid biosynthesis, all of which are necessary for the rapid proliferation typical of cancer cells. Furthermore, the pentose phosphate pathway produces reducing equivalents in the form of NADPH, which can be used to support lipid biosynthesis and regeneration of reduced glutathione. Reduced glutathione is used to counteract the effects of reactive oxygen species and other forms of oxidative damage. Compared to normal cells, cancer cells display increased oxidative stress [161–163] and are reliant upon antioxidant systems to prevent catastrophic damage that would lead to cell death [164]. Thus, dimeric PKM2 function effectively redirects the carbon flux away from the production of pyruvate and cellular energy but towards anabolic pathways required for rapid cell growth.

The greatly altered metabolism displayed by cancer cells is an attractive target for the development of various therapeutics and drugs combating cancer initiation and progression. Various treatment options are now being explored that specifically seek to modulate pyruvate metabolism to combat cancer. One of the best-known small molecule drugs is dichloroacetate [92]. Dichloroacetate inhibits the activity of all PDK isoforms resulting in the reactivation of the PDH complex, the increased consumption of pyruvate, and the decreased formation of lactate. Increased PDH activity, in turn, causes increased generation of ROS generated through aerobic respiration. In general, normal tissues are able to survive this increase. However, cancer cells are inherently pro-oxidative [161] and are unable to cope with the additional stress, which eventually leads to apoptosis [164, 165]. Studies in multiple cancer types, such as non-small cell lung carcinomas [166], squamous cell carcinomas [165], and breast carcinomas [167], have shown that upon dichloroacetate treatment cancer cell proliferation and tumor size decrease [168].

A second therapeutic target is LDHA. As discussed above, the generation of lactate is critically important to cancer cell metabolism and survival, both of which would be impaired by LDHA inhibition [169]. Furthermore, inhibition of LDH would increase the concentration of pyruvate within the cancer cell, which could then be metabolized in the mitochondria. Again, increased aerobic respiration would lead to increased ROS production, oxidative damage, and apoptosis. Specific inhibition of LDHA, and the dominant LDH isoform in cancer, is a clear goal in cancer research [28]. Fortunately, inhibition of LDHA should be well-tolerated in normal cells, as patients which are LDHA null have been described and display relatively mild symptoms [34].

Finally, given the strong dependence of cancer cells on glycolysis, directly impairing glycolysis may be a viable therapy. One such therapy is 2-deoxyglucose, a glucose analog which, upon phosphorylation by hexokinase, is unable to progress through glycolysis. 2-deoxyglucose is believed to compete with glucose at hexokinase, inhibit glycolysis, reduce intracellular ATP levels, and increase oxidative stress [170]. Additional hexokinase inhibitors are being studied as possible therapeutics to combat cancer. In many cancer cells, hexokinase 2, an embryonic isoform with limited expression in adult tissue, is highly upregulated in cancer [171, 172]. Inhibition or genetic ablation of hexokinase 2 is correlated with decreased tumor size and metastasis and increased life span [171]. A very promising drug, 3-bromopyruvate, has been shown to inhibit hexokinase, as well as other glycolytic enzymes, and causes ATP depletion in cancer cells, with complete eradication of the tumors possible [173]. One potential drawback to this strategy is the off-target effects on non-cancerous cell types that are highly reliant upon glycolysis for their energy needs, such as activated T-lymphocytes and astrocytes [174]. These inhibitors would reduce the excessive pyruvate production in cancer cells that spills over into lactate.

Interestingly, several key characteristics of cancer metabolism and the Warburg Effect are shared in Pulmonary Arterial Hypertension. Multiple etiologies can give rise to pulmonary arterial hypertension, though the symptoms are similar and are characterized by the constriction of the blood vessels in the lungs, which in turn increases blood pressure leading to hypertrophy and, eventually, heart failure [175, 176]. An interesting hallmark of Pulmonary Arterial Hypertension is the vascular remodeling that takes place in the muscle and epithelial tissues lining the constricted blood vessels. These cells display cancer-like characteristics including increased glycolytic metabolism, decreased oxidative phosphorylation, increased proliferation, and resistance to apoptosis [175, 177]. Indeed, many of the same factors previously mentioned in this review, such as HIF1 and PDK1, play important roles in the progression of pulmonary arterial hypertension [137].

### Neurodegeneration

The human brain is an incredibly complex and highly metabolic organ that is almost completely reliant upon glucose and pyruvate metabolism to generate cellular energy. Indeed, the brain accounts for 20–25 % of the body’s daily glucose consumption [178]. Ketone bodies may also be used, but only at significant levels during fasting [179]. Therefore, perturbations in glucose and pyruvate metabolism are expected to have striking neurological consequences. The severity of the neurological defect can be quite variable, correlating to some extent with the severity of the metabolic deficiency. Specific reports have described defects in PDH, MPC, PDP, and PC causing or associated with neurological disorders [1, 68, 89, 90, 113, 127, 130].

Altered or aberrant pyruvate metabolism is found in several major neurodegenerative disorders including Leigh’s syndrome, Alzheimer’s disease, and Parkinson’s disease [180]. Increased pyruvate levels in cerebrospinal fluid is considered a marker for Alzheimer’s disease [181], and a similar phenomenon has been observed in the blood serum of Parkinson’s disease patients [182]. Furthermore, Alzheimer’s disease patients typically display reduced PDH activity even though no change in PDH protein levels is observed compared to controls [183]. These observations indicate that deficits in CNS pyruvate metabolism contribute to or result from neurodegenerative disease.

#### Leigh’s syndrome

Nearly half of patients with PDH complex deficiency are diagnosed with Leigh’s syndrome [89, 90]. Historically, Leigh’s syndrome is characterized as a neurodegenerative disorder arising from deficiencies in the protein complexes associated with oxidative phosphorylation [184]. Patients with PDH complex deficiency learn to walk and crawl later, are less likely to interact with their environment, and respond slower to sensory stimuli than those with normal PDH function [89]. Leigh’s syndrome primarily affects the basal ganglia, thalamus, and brain stem where necrotic lesions form, leading to the loss of sensory and motor neurons and control [185]. In general, however, the prognosis for the majority of the patients is poor, with few surviving past the first decade of life [89].

#### Alzheimer’s disease

**Fig. 4:**
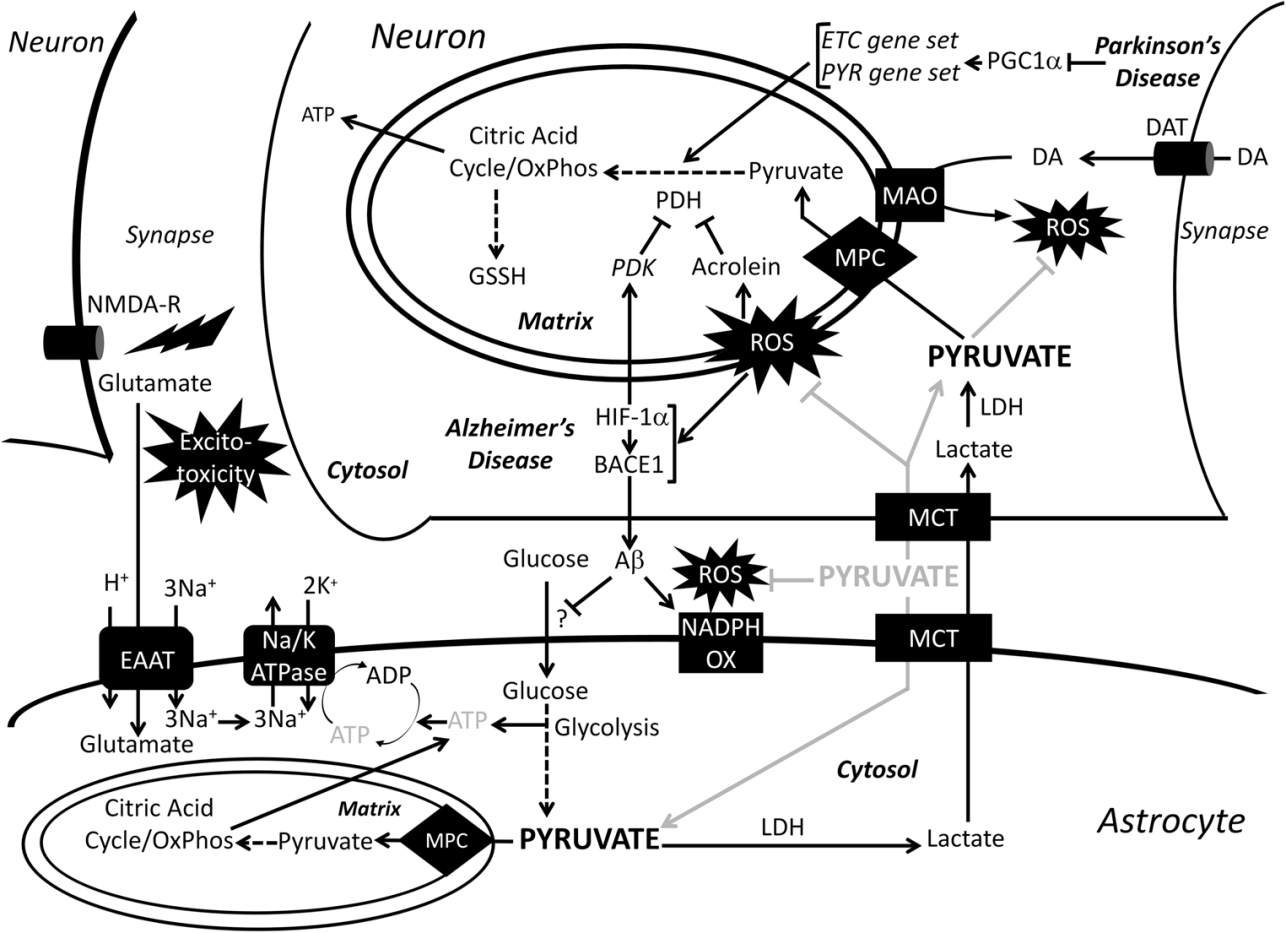
Role of pyruvate in neurodegeneration. Normal metabolic pathways of astrocyte and neuronal metabolism and pathological pathways of Alzheimer’s’s and Parkinson’s disease are drawn with *black arrows*. The *gray arrows* show the therapeutic pathway of exogenous pyruvate administration. In Alzheimer’s disease, ROS activates BACE1 and stabilizes HIF-1α, which further upregulates BACE1 levels. BACE1 produces Aβ, which has been shown to activate membrane-bound NADPH-oxidase leading to increased ROS production. HIF-1α also increases PDK levels leading to an inhibition of PDH. Peroxidation of lipids by ROS creates acrolein, which further inhibits PDH. Accumulation of Aβ has been shown to decrease glucose uptake by astrocytes, through a yet to be defined mechanism. The decrease in glucose uptake leads to a decrease in ATP fueling the Na/K ATPase, which is coupled to EAAT in order to clear glutamate from the synapse. The lingering glutamate in the synapse causes excitatory neurotoxicity. In Parkinson’s disease, the metabolism of DA by the outer mitochondrial membrane-bound MAO creates ROS. Furthermore, in Parkinson’s disease, PGC-1α, the transcription factor regulating election transport chain and pyruvate metabolism enzymes, is downregulated leading to decreased pyruvate metabolism. *MPC* mitochondrial pyruvate carrier, *PYR* pyruvate metabolism gene set, *NADPH OX* NADPH oxidase, *MAO* monoamine oxidase, *DAT* dopamine transporter, *DA* dopamine, *NMDA-R* NMDA receptor, *EAAT* excitatory amino acid transporter

Metabolic dysfunction plays a major role in the pathogenesis of Alzheimer’s disease (Fig. 4). A comprehensive review of normal brain metabolism is beyond the scope of this review; however, key points will be summarized here. Neurons rely heavily on oxidative metabolism and preferentially take up lactate, which is converted into pyruvate for oxidative phosphorylation by LDH, over glucose for their energy needs [186, 187]. Glucose, instead, is shuttled into the pentose phosphate pathway to create NADPH for regenerating reduced glutathione [188]. Astrocytes, on the other hand, are highly glycolytic cells that are responsible for storing glycogen in the brain, defense against oxidative stress, and maintenance of neuronal synapses and extracellular space homeostasis [189–191]. Astrocytes perform glycolysis and release lactate into the extracellular space, which is subsequently taken up by neurons in a process called the astrocyte–neuron lactate shuttle (ANLS). Thus, neuronal metabolism is dependent upon the uptake of lactate and its conversion to pyruvate by LDH to be used in the mitochondria for the generation of neuronal ATP. The ANLS is reviewed in greater detail by Bélanger et al. [192]. Recent research points towards ANLS as the critical energy supply for neurons [193, 194].

Several factors contribute to the metabolic dysfunction observed in Alzheimer’s disease, including the generation of reactive oxygen species (ROS), decreased glucose uptake, and synaptic hyper-excitability [195–200]. Production of ROS is considered to be one of the hallmarks in the pathogenesis of Alzheimer’s disease. Endogenously, ROS is produced by the mitochondria and by the membrane-bound enzyme NADPH oxidase [196, 201]. ROS species upregulate Beta-Site APP cleavage enzyme 1 (BACE1), which cleaves amyloid precursor protein (APP) into amyloid Beta (Aβ), leading to increased production of the Aβ oligomer, the peptide widely accepted as the main pathogen in Alzheimer’s disease. One way Aβ exerts neurotoxic effects is by increasing ROS production through activation of NAPDH oxidase [202]. Increased ROS, in turn, upregulates expression of hypoxia inducible factor-1α (HIF-1α) which stabilizes expression of BACE1 as well as increases the activity of PDK1, thereby reducing PDH activity and reducing mitochondrial pyruvate flux through oxidative phosphorylation [203]. Furthermore, peroxidation of lipids present in the brain creates a toxic product called acrolein, which is a potent inhibitor of lipoate-containing proteins, such as PDH [180, 204, 205]. An end result of neuronal ROS production is decreased PDH activity, which leads to decreased ATP production and neuronal dysfunction. The loss of proper neuronal function is absolutely central to the pathogenesis of Alzheimer’s disease. More recently, however, the role of astrocyte dysfunction has also been explored.

Astrocytes, as discussed earlier, employ a highly glycolytic metabolism. The network of regions in the brain where glycolytic metabolism is most prominent is called the default mode network (DMN). The DMN contains a high percentage of astrocytes [206] and is thought to play an important role in memory retrieval, and may be perturbed in Alzheimer’s disease [207]. While many questions about the pathogenesis of Alzheimer’s disease still exist, it is known that metabolic alterations in the DMN are significant enough to be detected by advanced FDG-PET imaging during the prodromal stages of Alzheimer’s disease [208–211]. A recent imaging study showed spatial correlation in the DMN between Aβ deposition and aerobic glycolysis in Alzheimer’s patients and cognitively normal individuals [212]. However, the difference in Aβ deposition between Alzheimer’s patients and normal individuals was greatest in regions where aerobic glycolysis was highest [212]. This raises the question of whether Aβ accumulation may lead to aerobic glycolysis as a protective or compensatory process for other metabolic abnormalities that accompany Alzheimer’s disease. Furthermore, another study found regional variations in the degree of overlap among Aβ deposits, hypometabolism, and atrophy in the brains of Alzheimer’s disease patients [213]. The lack of significant correlation between atrophy and hypometabolism may indicate the operation of region-specific pathological or protective mechanisms. Overall, greater induction of aerobic glycolysis may be a compensation for neuronal dysfunction and hypometabolism. This would increase lactate transfer by the ANLS, which is important for long-term memory formation and would also support neuronal oxidative metabolism by increasing pyruvate availability [214, 215].

#### Parkinson’s disease

Parkinson’s disease is the second most common neurodegenerative disease behind Alzheimer’s disease, and, like Alzheimer’s disease, mitochondrial dysfunction plays a significant role [216, 217]. Dopaminergic neurons in the substantia nigra are especially susceptible to oxidative damage because the mitochondrial enzymes involved in dopamine metabolism produce ROS (Fig. 4) [216, 218]. These neurons are killed by oxidative damage from the production of ROS and RNS (reactive nitrogen species) [216, 217]. The loss of these neurons leads to significant motor and non-motor neurological dysfunction that define the clinical course of Parkinson’s disease [219]. While oxidative damage has long been a focus of Parkinson’s disease, recent research suggests that hypometabolism is a significant contributor in the course of the disease. Recent studies have shown that, in the dopaminergic neurons of the substantia nigra of Parkinson’s disease and sub-clinical Parkinson’s disease patients, many genes regulating pyruvate metabolism and the electron transport chain are under-expressed [220]. These genes are under the control of PGC-1alpha, a transcription factor responsible for mitochondrial biogenesis and regulation, which is under-expressed in Parkinson’s disease. Over-expression of PGC-1alpha in a mouse model of Parkinson’s disease suppressed dopaminergic neuron loss [220]. This recent research could explain why hypometabolism can be seen on FDG-PET imaging in Parkinson’s disease patients just like in Alzheimer’s disease patients [221]. New research on Parkinson’s disease will continue to focus on the role of mitochondrial metabolism in pathogenesis and treatment [222].

#### Therapies

Alzheimer’s disease and Parkinson’s disease share many similarities: increased ROS production, hypometabolic states, and overall metabolic dysfunction in their respective regions of the brain. In Alzheimer’s disease, the two approved therapeutics are acetylcholine esterase inhibitors (AChE-I) and NMDA receptor inhibitors; however, these drugs only moderately improve cognition [223]. AChE-I increases cortical acetylcholine levels which are decreased in Alzheimer’s disease. NMDA receptor inhibitors block glutamate binding to the NMDA receptor, preventing neuronal excitatory toxicity. Parkinson’s disease patients are typically treated with l-Dopa, a dopamine pre-cursor, and inhibitors of dopamine catabolism [224]. None of the therapies listed address core problems of ROS and hypometabolism. However, administration of pyruvate has been shown to correct some of these central issues observed in neurodegeneration.

Generation of ROS is central to the pathogenesis of Alzheimer’s disease and Parkinson’s disease. Therapies that effectively alleviate oxidative stress are needed and evidence suggests that pyruvate administration may do so. Pyruvate is an endogenous scavenger of reactive oxidants hydrogen peroxide, superoxide, and peroxynitrite [225–228]. In cultured primary rat neurons, administration of pyruvate prevented Aβ-induced oxidative neuronal death [229, 230]. In a mouse model of Parkinson’s disease, the administration of ethyl pyruvate, an ethyl ester of pyruvate that is hydrolyzed into pyruvate and ethanol, protected substantia nigra neurons from oxidative neurotoxicity, which was attributed to metabolic protection provided by pyruvate metabolism [231]. Additional studies have shown that ethyl pyruvate administration inhibits RNS and ROS damage [232] and protects neurons from peroxide-induced damage [233]. A potential hypothesis explaining the protective effect of pyruvate relies on the oxidation of pyruvate and subsequent generation of NADH, which can be converted into mitochondrial NAPDH via NADP-transhydrogenase, which reduces ROS levels by replenishing reduced glutathione [234].

The development of a hypometabolic state, a key feature observed in Alzheimer’s disease and Parkinson’s disease, may be corrected by the administration of pyruvate. In one study, exogenous administration of pyruvate and 3-beta-hydroxybutyrate directly into the cerebrospinal fluid was shown to ablate excitatory neurotoxicity and corrected neuronal energy deficiency in a mouse model of Alzheimer’s disease [203]. In astrocytes, the ATP generated by aerobic glycolysis is used to fuel glutamate uptake by excitatory amino acid transporter [192, 235, 236]. However, when astrocyte glucose uptake is impaired, astrocytes lack the energy to sufficiently clear glutamate from the synapse. This leads to excitatory neurotoxicity, defined in part by increased ROS and mitochondrial dysfunction, and neuronal death [237]. Astrocytes in mice fed pyruvate and 3-beta-hydroxybutyrate had twice the glycogen stores compared to standard diet controls. Pyruvate and 3-beta-hydroxybutyrate were thought to provide a non-glucose energy source that spared the use of astrocyte glycogen enabling maintenance of synaptic homeostasis [203].

Given pyruvate’s role in mitochondrial metabolism and its anti-oxidant capabilities, therapeutics modulating pyruvate metabolism may be a fruitful area for future study in the treatment for Alzheimer’s disease and Parkinson’s disease (Fig. 4). Modulation of pyruvate metabolism may also be beneficial in other neurodegenerative diseases with mitochondrial dysfunction such as progressive supranuclear palsy, a disease that has pathophysiology similar to Alzheimer’s disease and Parkinson’s disease [238, 239]. Increasing CNS pyruvate metabolism looks like a promising neuroprotective therapy, but more research and trials need to be carried out to establish methods of successfully delivering pyruvate to the CNS and mitochondria of affected cells.

### Heart failure

Heart failure is a condition defined as the inability of the heart to adequately supply oxygen and nutrients via the blood to the tissues of the body. This disorder affects approximately 2 % of the US population and increases to 6–10 % of people over age 65, representing an incredible burden on the US healthcare system [240, 241]. Patients diagnosed with heart failure display symptoms including, fatigue, weakness, confusion, and increased heart rate. The leading causes of heart failure include myocardial infarction and hypertension [241, 242]. These injurious events alter the physiology of the heart resulting in changed gene and protein expression patterns [243–245]. In response to these changes, the heart fails to produce enough energy to meet its large energetic demand. Indeed, patients suffering from heart failure have decreased ATP and phosphocreatine levels [240, 246].

Many main-stay therapies aim to reduce the workload of the failing heart [247]. A complementary approach, however, is to modulate cardiac metabolism so that the heart produces more energy with the supplies available [248–250]. Under normal conditions, the heart obtains the majority of its energy through the beta-oxidation of fatty acids. However, beta-oxidation of fatty acids is an inefficient fuel source, requiring greater amounts of oxygen per ATP produced as compared to the oxidation of glucose and pyruvate [243, 251]. Beta-oxidation creates acetyl-CoA, which is consumed through the citric acid cycle and oxidative phosphorylation. Oxidation of glucose and pyruvate, however, generates ATP by both substrate level and oxidative phosphorylation, generating more ATP per oxygen consumed. Based on the predicted number of ATP molecules produced per oxygen atom reduced when utilizing glucose or fatty acids as an exclusive fuel source, cardiac efficiency on fatty acids would be near 10 % less [248, 252]. However, as empirically observed, due to unknown mechanisms, this efficiency decrement approaches 30 % [248, 252]. Thus, therapies that increase relative amounts of glucose oxidation might substantially increase cardiac efficiency and therefore energy reserves and longevity.

These therapies include drugs which either inhibit or downregulate the enzymes associated with beta-oxidation [242, 248, 253, 254]. Alternatively, glucose and pyruvate oxidation can be increased directly. Inhibition of PDK, by dichloroacetate, for example, will relieve the negative regulation on PDH, thereby increasing PDH activity and glucose oxidation [255]. Indeed, dichloroacetate has been shown to confer cardioprotective effects and increase cardiac efficiency in rat hearts [248, 256, 257].

### Additional disorders of pyruvate metabolism

Appropriate regulation of pyruvate flux is critical for maintaining cellular function in multiple contexts. Pyruvate dysmetabolism because of excessive inhibition of PDH by PDK presents in chronic, progressive diseases such as chronic obstructive pulmonary disease (COPD), obesity, diabetes, and aging.

Patients with chronic obstructive pulmonary disease have impaired skeletal muscle capacity to generate ATP leading to exercise intolerance [258–261]. This ATP deficit is caused by a decrease in oxidative skeletal muscle fibers and a decrease in citric acid cycle carbon flux in skeletal muscle fibers [258–261]. Clinical trials of dichloroacetate to upregulate pyruvate metabolism during chronic obstructive pulmonary disease have found improved exercise tolerance [262, 263]. The positive results from dichloroacetate clinical trials indicate that misregulation of PDH may play a role in the pathogenesis of chronic obstructive pulmonary disease.

Patients with obesity or type 2 diabetes often have impaired regulation of carbohydrate metabolism concomitant with mild exercise intolerance [264, 265]. Metabolic inflexibility is the inability to properly switch from fat to carbohydrate oxidation either post-prandial or during exercise, and is often present in obesity and type 2 diabetes [266, 267]. Increased serum and intramuscular lipids increase PDK activity and thereby reduce PDH activity and pyruvate flux into the citric acid cycle [268, 269]. Furthermore, in healthy subjects, a high-lipid, low-carbohydrate diet leads to impaired PDH activity via PDK upregulation [270]. Several studies have shown that pyruvate combined with exercise can restore PDH activity [271–273]. Pyruvate supplementation combined with moderate physical activity leads to significant decreases in body weight and fat mass and a significant increase in exercise tolerance [274]. Thus, restoring normal pyruvate metabolism may relieve major aspects of the metabolic pathology present in type 2 diabetes and obesity.

Pyruvate dysmetabolism also contributes to failure of the pancreatic islet β-cells during late type 2 diabetes. In diabetic mouse and rat models, islet β-cell PDH activity is severely impaired by increased PDK activity [275, 276]. Furthermore, PC activity is also decreased in the islet β-cells of diabetic mice [277]. PC plays a critical role in islet cell proliferation [278] and increased insulin secretion in compensation for whole-body insulin resistance [279]. Inactivation of PC is thought to be involved in the transition from mild hyperglycemia to severe hyperglycemia [277].

Pyruvate dysmetabolism is also seen in many organs in diabetes. In the heart, PDK4 is upregulated leading to excessive fatty acid oxidation and ROS formation in the mitochondria [280]. Muscle, liver, and kidney are among other tissues are severely affected by diabetes. In diabetic skeletal muscle and liver, PDK is expression is increased, leading to PDH inhibition [281, 282]. In diabetic kidneys, PDH is inhibited by preferential oxidation of fatty acids leading to increased ROS production [283, 284]. In diabetic rat kidneys, the administration of ethyl pyruvate protected against diabetic nephropathy, regardless of blood glucose levels [285]. Pyruvate administration protected against cataract formation and increased cellular ATP levels in a mouse model of diabetes [286]. Pyruvate administration also prevented zinc-induced islet β-cell death in a mouse model of diabetes by protecting cellular ATP levels [287]. Recent studies have also shown that altered pyruvate metabolism is involved in the aging process. In the aging mouse brain, there is an increase in lactate caused by diminished pyruvate flux through the citric acid cycle [288]. Two studies in *Caenorhabditis elegans* concluded that a long lifespan is dependent on PDH activity, noting that inhibition of PDH reduced lifespan [289] and that inhibition of PDK increased lifespan [290].

Misregulation of the PDK–PDH axis may also result from a genetic mutation in a regulatory gene. Subjects with Chuvash Polycythemia have a mutated form of the Von Hippel Lindau protein, preventing it from properly binding HIF-1α and targeting it for degradation. As with cancer, HIF-1α accumulates, thereby increasing transcription and activity of PDK. As occurs elsewhere, increased PDK activity decreases PDH activity and pyruvate flux through the citric acid cycle [291]. This mechanism is thought to be central to the exercise intolerance experienced by Chuvash Polycythemia subjects [292, 293]. Tests performed on Chuvash Polycythemia patients showed increased lactate production during exercise and reduced exercise capacity, as well as significant increases in mRNA of PDK in skeletal muscle, elevated blood pyruvate, and elevated blood lactate, compared to controls.

## Conclusion

The regulation of pyruvate metabolism in humans is highly complex, involving several major enzymes, many encoded by multiple genes and comprising numerous individual protein subunits. Mutations in any of these genes and disruption of pyruvate metabolism at any of these major nodes may lead to disease. New aspects of pyruvate metabolism are being continuously discovered and will lead to greater delineation between healthy verses pathological pyruvate flux. For example, the recent discovery of the MPC elucidated the molecular basis for metabolic disease in two patient families [1, 2]. In addition to targeted approaches to specifically modulate defective aspects of pyruvate metabolism, major nodes of pyruvate metabolism may be up- or downregulated to treat disease in a compensatory or secondary manner. Indeed, specific inhibition of the MPC was recently shown to increase skeletal muscle insulin sensitivity by activating the AMP-activated protein kinase pathway [63]. Because most major diseases also involve aberrant metabolism, understanding and exploiting pyruvate carbon flux may yield novel treatments that will enhance human health.

## Abbreviations

Aβ: Amyloid beta
ALT: Alanine aminotransferase
ATP: Adenosine triphosphate
ADP: Adenosine diphosphate
AMP: Adenosine monophosphate
ANLS: Astrocyte-neuron lactate shuttle
CoA: Coenzyme A
E1: E1 subunit of PDH
E2: E2 subunit of PDH, also known as dihydrolipoyl acetyltransferase
E3: E3 subunit of PDH, also known as dihydrolipoyl dehydrogenase
E3BP: E3 binding protein
FAD: Flavin adenine dinucleotide
FADH_2_: Reduced form of FAD
GDP: Guanosine diphosphate
GTP: Guanosine triphosphate
HIF1: Hypoxia inducible factor 1
LDH: Lactate dehydrogenase
MPC: Mitochondrial pyruvate carrier
NAD^+^: Nicotinamide adenine dinucleotide
NADH: Reduced form of NAD^+^
PC: Pyruvate carboxylase
PDH: Pyruvate dehydrogenase
PDK: Pyruvate dehydrogenase kinase
PDP: Pyruvate dehydrogenase phosphatase
PEPCK: Phosphoenolpyruvate carboxykinase
PGC-1α: PPARγ coactivator 1α
Pi: Phosphate ion
PK: Pyruvate kinase
ROS: Reactive oxygen species
